# Optimizing Root Canal Therapy: An In Vitro Comparative Study of Innovative File Systems on Mandibular Premolar Fracture Resistance

**DOI:** 10.7759/cureus.62077

**Published:** 2024-06-10

**Authors:** Shruthi P, Jaya Nagendra Krishna M, Jayaprada Reddy, Nikhil Kumar N, Dilip Jayyarapu, Rajani Punna, Divya R, Anuhya S

**Affiliations:** 1 Conservative Dentistry and Endodontics, Kamineni Institute of Dental Sciences, Nalgonda, IND; 2 Prosthodontics, Kamineni Institute of Dental Sciences, Nalgonda, IND

**Keywords:** xp - endo shaper, self adjusting file, reciproc, mandibular premolars, fracture resistance

## Abstract

Introduction and aim: Root canal therapy is a vital procedure for saving teeth by removing infection and cleaning the complex root canal system. However, a delicate balance exists between thorough cleaning and preserving tooth strength. The study aims to evaluate the instrumentation effect of three innovative file systems, XP-endo® shaper, Reciproc®, and Self-adjusting file (SAF) on fracture resistance of mandibular premolars.

Materials and methods: Thirty single-rooted mandibular premolars were collected; a standard access cavity was prepared and the working length was established 1 mm short of the apex. The teeth were randomly divided into three groups(n=10). In Group 1, the shaping of the specimens was achieved using XP-endo® shaper; in Group 2, it was instrumented using Reciproc® file; and in Group 3, it was instrumented using SAF. All samples were decoronated and the roots were mounted vertically in acrylic resin and subjected to fracture resistance under a universal testing machine.

Results: Intergroup analysis was done by one-way ANOVA followed by Bonferroni post hoc test, which did not report a statistically significant difference (p>0.05).

Conclusion: All three tested file systems were similar in fracture resistance. XP-endo® shaper exhibited better fracture resistance on root canal instrumentation when compared to Reciproc® and SAF although they are not statistically significant.

## Introduction

Root canal therapy is a vital procedure for saving teeth by removing infection and cleaning the complex root canal system. However, a delicate balance exists between thorough cleaning of root canal system and preserving tooth strength. Traditional methods and excessive removal of dentin can increase the risk of vertical root fractures - a major complication often leading to tooth loss [1].

Endodontic treatment, while preserving teeth, can impact their structural integrity. Studies have shown an increased risk of fracture in treated teeth compared to untreated ones. Therefore, minimizing dentin removal during the procedure is crucial to optimizing post-treatment tooth strength [2]. Loss of moisture content within the dentin has been theorized as a contributing factor to the increased fracture susceptibility observed in endodontically treated teeth [3]. 

This study explores advancements in root canal shaping techniques to improve tooth preservation. The use of motorized nickel-titanium (NiTi) files for root canal preparation may contribute to dentin weakening and potentially increase the risk of vertical root fractures (VRFs) [1]. Technological advancements have led to rapid evolution in canal preparation techniques in recent years. The introduction of rotary nickel-titanium (NiTi) instruments for canal preparation has changed canal shape, size, and taper as compared to hand instrumentation [4]. To address this, innovative NiTi file systems have been developed, aiming to effectively clean canals while minimizing dentin removal [5]. Studies by Kim et al. suggest a possible association between nickel-titanium (NiTi) file design and the incidence of longitudinal root fractures (LRFs). This research investigates the fracture resistance of teeth treated with three such systems, paving the way for safer and more effective root canal therapy [6].

A new wave of root canal instrumentation systems touts "3D instrumentation" or "anatomical cleaning and shaping." These systems utilize heat-treated files that can switch between martensite and austenite phases. This allows the files to conform to the unique curves of each root canal, enhancing their super-elasticity and shape memory during treatment. This translates to less unnecessary dentin removal, reduced debris extrusion, and a minimized risk of microcrack formation [5].

The XP-endo® shaper system offers a novel approach to root canal shaping. This system addresses the demand for more efficient three-dimensional (3D) cleaning and minimally invasive procedures. The instruments are likely characterized by favorable properties such as superelasticity, good thermal adaptation, resistance to fatigue, and the ability to withstand rotational pressures [7]. Reciproc® files have an S-shaped cross-section with two cutting edges, with variable taper, which is fixed 3 mm from the apex of the files and decreases in the middle and coronal thirds [8].

The Self-adjusting File (SAF) is a novel instrument designed for root canal preparation. This hollow, flexible file boasts the ability to conform to the unique anatomy of each canal. Compared to rotary and reciprocating files, SAF technology may address a greater proportion of irregularly shaped canals. Additionally, SAF use might contribute to a cleaner dentin surface within the root canal, potentially facilitating successful three-dimensional obturation. Studies suggest that teeth instrumented with SAF may exhibit reduced crack formation and improved resistance to fracture [9].

The core objective of endodontic treatment is to preserve natural teeth by employing safe and effective techniques. However, the mechanical procedures used during root canal preparation can potentially introduce microscopic cracks (microcracks) within the dentin. These microcracks may propagate into full root fractures, ultimately compromising the long-term success of the treatment. For this reason, rigorous safety assessments are critical for any newly developed endodontic file [10]. This investigation aimed to comparatively evaluate the fracture resistance of mandibular premolars following root canal preparation using three innovative file systems: XP-Endo® Shaper, Reciproc®, and Self-adjusting files.

## Materials and methods

A power analysis was conducted using G*Power 3.1 software (Heinrich Heine Universität Düsseldorf, Düsseldorf, Germany) to determine the appropriate sample size. The analysis indicated that a sample size of 30, with 10 participants per group, would be sufficient to achieve 90% power to detect an effect size of 0.7 at a significance level of 5%.

For this study, intact human mandibular premolars were obtained. Each tooth possessed a single root canal with a fully developed apex. The teeth were stored in a saline solution mimicking saliva and maintained at 37°C until use. Prior to experimentation, all samples underwent examination under a stereomicroscope at 10X magnification to identify any pre-existing defects such as craze lines or cracks. Teeth exhibiting such flaws were excluded from the study. Following the outlined procedure, we selected a total of 30 samples for inclusion in the study. Standard access cavity preparation was done with an airotor handpiece using Endo access cavity preparation bur. The working length of each canal was visually established by placing a size 10 K hand file until the tip of the file was visible at the tip of the apical foramen. The working length was established 1 mm short of the apex and verified with RVG. Teeth were then randomly divided into Three groups of 10 each based on the type of file system employed. Biomechanical preparation was done initially using 10 and 15 k-files and then with XP-endo® shaper (Group 1), Reciproc® (Group 2), and Self-adjusting file (Group 3).

**Figure 1:**
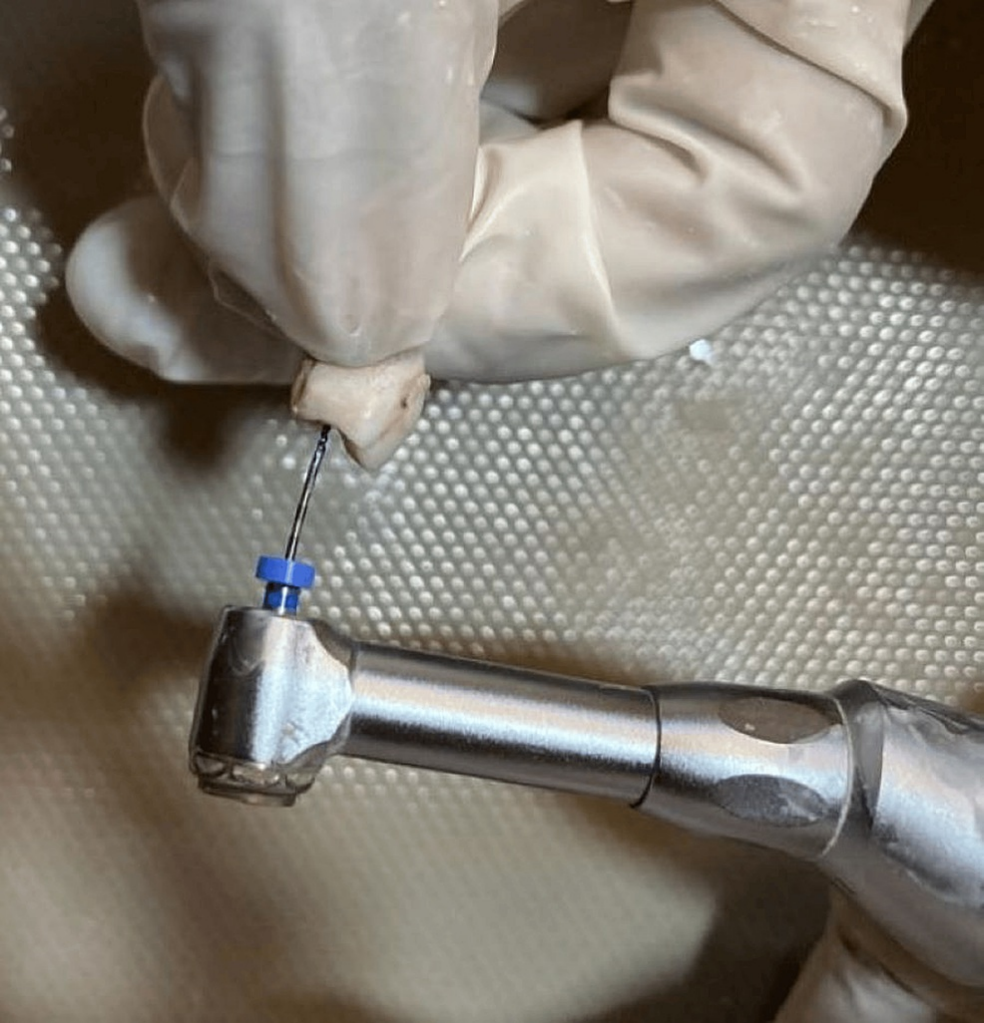
Instrumentation of mandibular premolar with XP-endo® shaper XP-endo® shaper (FKG Dentaire, Switzerland)

Group 1: The root canal shaping procedure was performed using a commercially available single-file system (XP-endo® shaper, FKG Dentaire, Switzerland) according to the manufacturer's guidelines. Following the creation of a glide path with a #15 k-file (Dentsply Maillefer, Switzerland), a single new file was used per tooth to prepare the canal to a working length and size #30 with a 0.04 taper (Figure 1).

**Figure 2:**
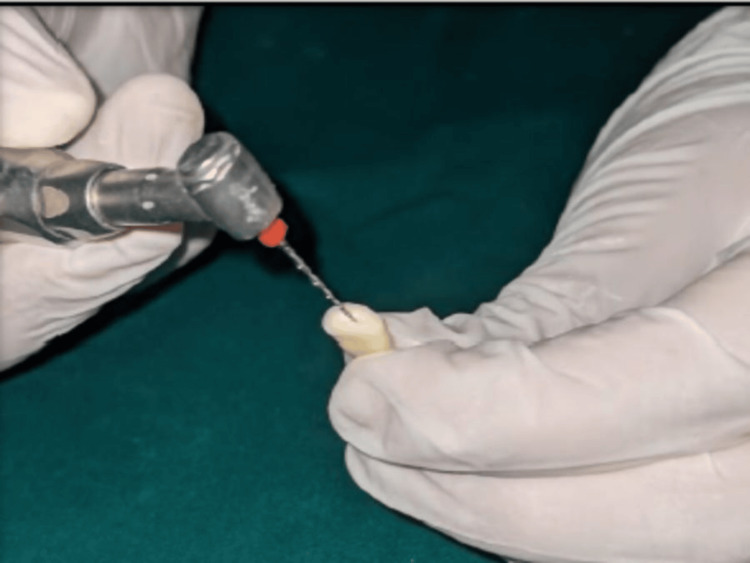
Instrumentation of mandibular premolar with Reciproc® Reciproc® file (VDW Dental, Munich, Germany)

Throughout the shaping process, the canal was irrigated with 3 ml of 5.25% sodium hypochlorite (NaOCl) solution. Group 2: The root canals in this group were shaped using a Reciproc® file (VDW Dental, Munich, Germany) with a variable taper. The instrumentation was performed at a speed of 300 rpm and a torque of 5 N.cm using an X-Smart endomotor (Dentsply Sirona, USA). The file was moved in a reciprocating and pecking motion (in and out) throughout the canal length to achieve a final preparation size #25 with a 0.08 taper (Figure 2).

**Figure 3:**
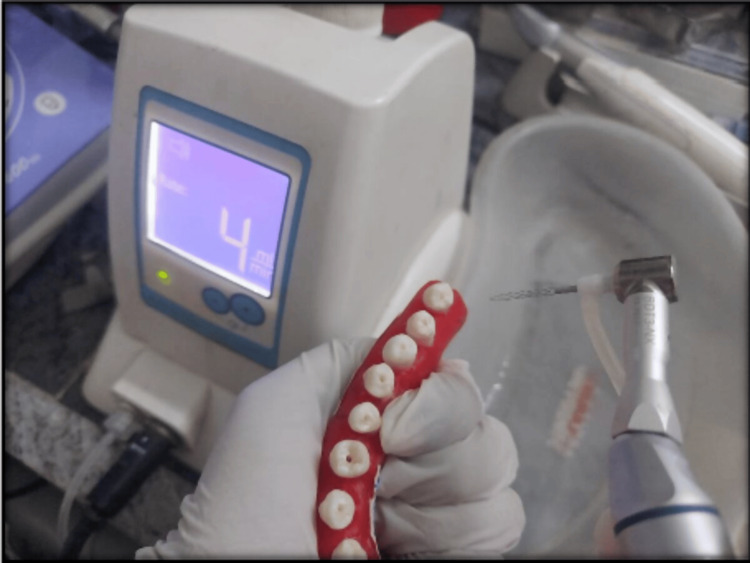
Instrumentation of mandibular premolar with SAF SAF: Self-Adjusting File (ReDent Nova, Berlin, Germany)

Following shaping, the canals were irrigated with 3 ml of 5.25% sodium hypochlorite (NaOCl) solution. Group 3: The root canals were prepared using a 1.5 mm Self-adjusting file (SAF) with a translinear motion (in and out). The SAF instrument was operated in a pecking motion at a speed of 5000 vibrations per minute (vpm) within an Endo station endodontic system (ReDent Nova, Berlin, Germany) (Figure 3).

The instrumentation process lasted 4 minutes with continuous irrigation using a 5.25% sodium hypochlorite (NaOCl) solution at a flow rate of 4 ml/min, as recommended by the manufacturer. The irrigation solution was delivered through the hollow core of the SAF file via an integrated peristaltic pump.

Final irrigation protocol (Groups 1-3)

Following instrumentation, a final irrigation sequence was performed for all groups. This involved a 1-minute flush with 5 mL of 17% aqueous EDTA (Dent Wash, Prime Dental Products) to remove the smear layer, followed by a 1-minute rinse with 5 mL of 5.25% NaOCl solution. The final rinse consisted of 5 mL of distilled water.

Root segment preparation and mounting

To obtain root segments for biomechanical testing, all samples were sectioned at the cementoenamel junction using a diamond-coated bur with continuous water cooling. These root segments were then placed upright in standardized cylindrical molds filled with a special acrylic material that hardened on its own. Each root was placed in the center of the mold and held in place with a thin layer of wax (0.2 to 0.3 mm thick) before the acrylic hardened. Once the material had solidified, the roots were taken out, and any remaining wax was meticulously removed. To mimic the tissue surrounding the tooth (periodontal ligament), a thin layer of silicone-based material (polyvinyl siloxane) was applied inside the root cavity within the acrylic mold. Finally, the roots were put back in the molds.

Biomechanical testing

**Figure 4:**
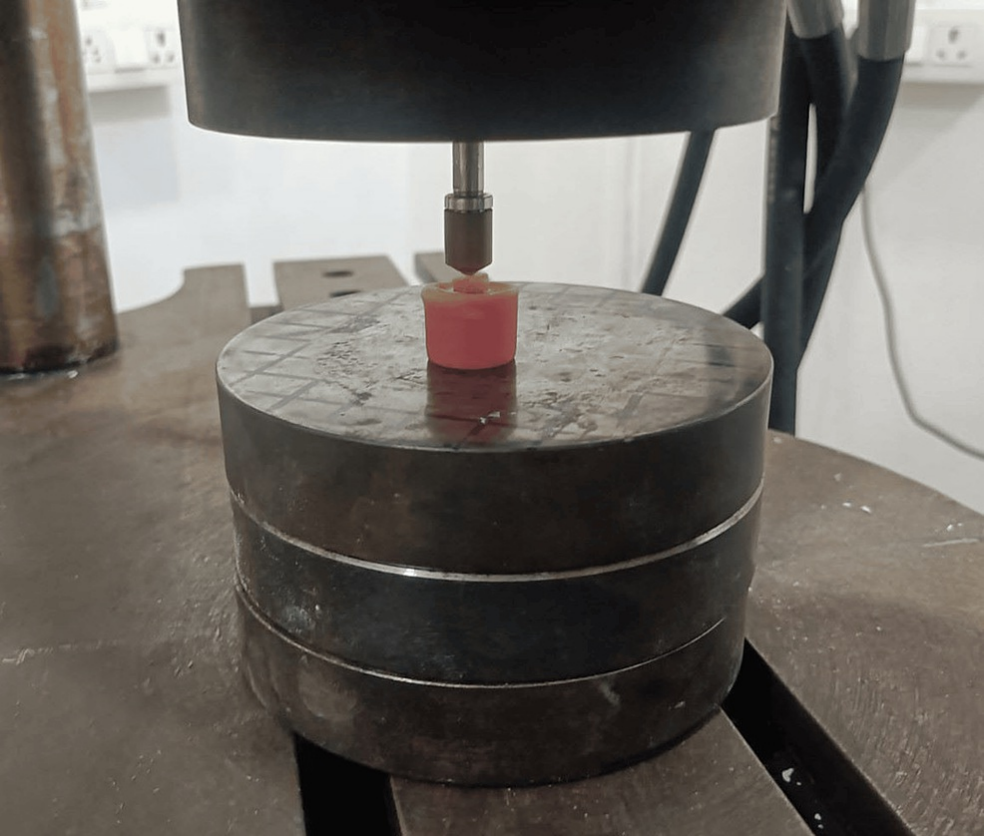
Acrylic blocks placed on the universal testing machine for fracture resistance Universal testing machine (Instron Corp, Canton, MA)

The prepared acrylic blocks containing the mounted roots were secured on a universal testing machine (Instron Corp, Canton, MA). A small round tip, 3 mm in diameter, was placed directly above the opening of the canal in each root segment (canal orifice). A steady downward force was then applied at a speed of 1 mm per minute until the root broke (Figure 4). The maximum amount of force (in Newtons, denoted by N) needed to break each sample was recorded.

## Results

Statistical analysis

Data was analyzed using SPSS software, version 26.0 (IBM Corp., Armonk, NY)) and the level of significance was set at p<0.05. Descriptive statistics were performed to assess the mean and standard deviation of the respective groups (Table 1). The normality of the data was assessed using the Shapiro-Wilkinson test. Inferential statistics to find out the difference within the group was done using one-way ANOVA followed by the Bonferroni post hoc test.

Results

Shapiro-Wilkinson test for normality reported a significant difference(p<0.05), Hence, Non-Parametric tests are used for the analysis. Regarding ‘FRACTURE RESISTANCE’. Between-group analysis by the Kruskal Wallis test did not report a significant difference (p>0.05).

**Table 1:** Comparison of fracture resistance of Premolars *P<0.05 is statistically significant (Shapiro Wilkinson test, p<0.05) SAF: Self-adjusting file (ReDent Nova, Berlin, Germany); XP-endo® shaper (FKG Dentaire, Switzerland); Reciproc® file (VDW Dental, Munich, Germany).

		Mean	SD	Mean Rank
GROUP 1(XP-endo® shaper)		2228	469.83	9.8
GROUP 2(Reciproc®)		1896	327.03	6.3
GROUP 3(SAF)		2032	520.71	7.9
P-value (Kruskal Wallis test)		0.46		
P-value (Boneferroni) posthoc test	G 1 vs G2	0.48		
	G 1 vs G3	0.77		
	G2 vs G3	0.88		

## Discussion

The development of nickel-titanium (NiTi) alloys has significantly transformed the field of root canal preparation over the past two decades. These instruments offer enhanced flexibility compared to their predecessors, attributable to a combination of their low elastic modulus and high torsional resistance [11]. Beyond facilitating efficient canal cleaning (debridement), rotary NiTi systems achieve superior canal wall cleanliness due to a confluence of factors. These factors include increased instrument tapers, diverse file designs, advancements in metallurgy, and optimized rotational motion. However, the increased taper of these instruments raises concerns about the potential over-removal of dentin, a crucial component of tooth structure [12].

Vertical root fractures (VRFs) are a major concern in endodontics, posing a significant clinical challenge due to their difficulty in diagnosis and limited treatment options [13]. The increasing prevalence of VRFs in endodontically treated teeth necessitates a focus on minimizing iatrogenic factors during root canal procedures [14].

While endodontists strive to save natural teeth with safe tools and methods, mechanical cleaning of root canals (root canal preparation) can create tiny cracks that might grow into major fractures, jeopardizing the tooth's long-term health. Therefore, evaluating the safety of any new instrument for this procedure is crucial [15,16]. Root canal instrumentation using the rotary and reciprocating files reduces the fracture resistance of the instrumented tooth by up to 30% [17]. It has been previously established that the SAF exhibits better fracture resistance after instrumentation compared to rotary and reciprocating NiTi files [1].

The instrument systems used in this study were XP-endo® shaper, Reciproc®, and SAF systems, each used with a full sequence of instruments as recommended by their manufacturers and the traditional manual instrumentation using K-files. To assess the fracture resistance of the prepared root canals, a vertical load was continuously applied until fracture occurred. In this experiment, the canals were intentionally left unfilled to isolate the impact of the instrumentation procedures on the forces required for fracture initiation [18].

This study investigated the impact of various instrumentation techniques on the fracture resistance of mandibular premolars. Our findings demonstrated that the XP-endo® shaper (XP) system exhibited superior fracture resistance compared to Reciproc® and Self-adjusting file (SAF) systems.

The XP system utilizes a snake-shaped NiTi instrument constructed from MaxWire, a unique martensitic-austenitic-electropolished thermomechanically treated NiTi alloy [19]. This material undergoes a phase transformation at body temperature, enabling the instrument to curve and adapt to the complexities of the root canal anatomy. The manufacturer emphasizes the benefits of its flexibility and preset shape, allowing the XP to navigate intricate canals while minimizing dentin removal through its 0.01 taper and ISO size 30 diameter. The tip of the instrument has six blades (Booster tip) that can start shaping the canal after a manual glide path is created (at least size 15 ISO). The XP system gradually increases the size of the canal to a final size of ISO 30 with a taper of 0.04 [20]. Recent studies support these claims, suggesting improved performance in challenging canals with minimal impact on dentin walls. This potentially reduces the formation of microcracks, ultimately contributing to increased fracture resistance [21]. Studies suggest that nickel-titanium (NiTi) files with specific properties, such as those incorporating M-wire or controlled memory technologies, may exhibit a lower incidence of crack formation within the root canal dentin. This potentially arises from their enhanced elasticity compared to traditional NiTi files [22]. Our investigation revealed that the XP-endo® shaper file, potentially due to its increased elasticity compared to other instruments, exhibited a lower incidence of crack formation within the dentin and demonstrated superior resistance to fracture.

Studies by Wilcox et al. have shown a link between increased tooth structure removal and a higher risk of root fractures. Instruments with larger tapers remove more dentin, which can make the root more susceptible to fracture [23]. Building on previous research, this study aimed to investigate the impact of instrumentation on microcrack formation. Bayram et al. compared the effects of XPES and ProTaper Gold (PTG) systems on microcrack development. Their findings indicated a statistically significant increase in microcrack incidence within dentinal structures following PTG instrumentation, whereas the XPES system did not appear to induce new microcracks [24]. 

Contributing to the understanding of vertical root fractures, Kim et al. investigated the potential link between nickel-titanium (NiTi) instrument design and fracture likelihood. Their study suggests that stiffer NiTi file designs may lead to higher stress concentrations within the apical dentin of curved canals during shaping procedures. This increased stress concentration could potentially elevate the risk of crack formation [6].

The SAF system, designed for minimally invasive canal shaping, utilizes a hollow, thin-walled nickel-titanium lattice with an abrasive outer surface [25]. The Self-adjusting file (SAF) employs an abrasive surface to facilitate the removal of a minimal, uniform layer of dentin along the entire canal wall. This controlled approach aims to preserve the integrity of the surrounding dentin structure [1].

Marco Aurelio Versiani et al. (2011) reported increased canal volume and prepared wall percentages with SAF compared to rotary instrumentation in the coronal third, concerns exist regarding dentin integrity. The abrasive nature of the SAF might contribute to the loss of pericervical dentin, potentially weakening the tooth structure and potentially influencing fracture resistance [26]. A study by Liu et al. investigated the impact of different file systems, Reciproc, One-Shop, ProTaper, and SAF in terms of dentin cracks.. Their findings indicated that SAF and Reciproc instruments resulted in fewer cracks compared to ProTaper and One-Shop files [27]. In the present study, the XP-endo® shaper system exhibited superior fracture resistance than SAF. Reciprocating files, while offering advantages, have been associated with the formation of microcracks within the dentin, possibly due to their reciprocating motion [8]. This aligns with our observations of potentially reduced fracture resistance in the Reciproc® group, although statistically insignificant.

This study investigated how different root canal instrumentation techniques (XP-endo® shaper, Reciproc®, and SAF) affect the fracture resistance of mandibular premolars. Focusing on a critical concern in endodontics, maintaining tooth strength during root canal procedures, the research assessed the impact of various file systems on this vital factor for long-term treatment success. The study employed standardized methods for sample selection, canal preparation, and fracture resistance testing. This approach strengthens the study's internal validity and ensures a fair comparison between the groups. While statistically significant differences were not observed (p>0.05), a trend emerged where the XP-endo® shaper group exhibited the highest mean fracture resistance compared to the Reciproc® and SAF groups. This trend aligns with the discussion about the XP system's potential advantages in preserving dentin and minimizing microcrack formation.

Clinical significance

While this study didn't find statistically significant differences, the XP-endo® shaper system showed promise in preserving tooth structure compared to other techniques. This is clinically significant because saving tooth structure during root canals is essential for long-term tooth strength and reduces the risk of fractures. If further research confirms these findings, the XP-endo shaper® system could potentially lead to improved clinical outcomes and a more minimally invasive approach to root canal therapy.

Limitations of the study

This in-vitro study utilized a limited sample size and did not account for the complexities of the clinical environment. Further research is warranted to investigate the long-term clinical performance of the XP system and compare it to other instrumentation techniques in a more realistic setting. Additionally, incorporating cyclic fatigue testing could provide further insights into the influence of these techniques on fracture resistance under simulated functional loading.

## Conclusions

The XP-endo shaper® system demonstrated promising results in terms of preserving fracture resistance in mandibular premolars compared to Reciproc® and SAF systems. The unique properties of the MaxWire alloy and the design features of the XP system might contribute to these findings. Further clinical research is recommended to validate these observations and assess the long-term clinical efficacy of the XP system.
